# Excision primary anastomosis urethroplasty with gracilis muscle flap for management of complex stricture with prostatorectal fistula and failure of multiple surgeries: A case report

**DOI:** 10.1016/j.eucr.2025.103238

**Published:** 2025-10-08

**Authors:** Kristian Mohamad Daniputra, Paksi Satyagraha, Pradana Nurhadi

**Affiliations:** Department of Urology, Universitas Brawijaya Faculty of Medicine, Saiful Anwar General Hospital, Jaksa Agung Suprapto 2, Klojen, Malang, Jawa Timur, 65112, Indonesia

**Keywords:** Complex urethral strictures, EPA urethroplasty, Gracilis muscle flap

## Abstract

Complex urethral strictures are challenging to manage, particularly after failed urethroplasty and associated complications. We report a case of a 32-year-old male with a nine-year history of urinary retention following pelvic trauma and six prior surgeries, including an iatrogenic prostatorectal fistula. The patient underwent re-do excision and primary anastomotic (EPA) urethroplasty combined with gracilis muscle flap interposition for fistula repair. Inferior pubectomy and bulbar urethra transection were performed. Postoperative recovery was uneventful, and uroflowmetry showed satisfactory flow (Qmax: 14.8 mL/s). EPA urethroplasty with gracilis flap may improve outcomes in complex urethral stricture with fistula.

## Highlights

• EPA urethroplasty was used to manage complex stricture after multiple failed surgeries.
• Gracilis muscle flap provided vascular support and fistula coverage.
• Inferior pubectomy enabled tension-free urethral anastomosis.
• Postoperative Qmax was 14.8 mL/s with six-month follow-up.
• This case supports gracilis flap use in complex urethral repairs.

## 1 Introduction

Pelvic fracture-related urethral injury (PFUI) typically results from high-impact trauma such as motor vehicle accidents or falls. Approximately 10 % of pelvic fractures involve urethral injury, with pubic extension or displacement being common contributors.^1^ Pelvic fractures have an incidence of 20/100,000 in men and 29/100,000 in women,^2^ and up to 90 % of cases are accompanied by other injuries, including PFUI, which carries a mortality risk of 5–33 %.^3^ Fractures involving all four pubic rami or pelvic ring disruption are frequently associated with urethral injury.^2^

Complex urethral strictures include long-segment (>4 cm) lesions, strictures following failed urethroplasty, multiple failed hypospadias repairs, and those due to pelvic fractures. The incidence of complex urethral strictures from pelvic fracture is reported at 13.8 %.^4^

PFUI can be initially managed either with primary endoscopic realignment (PER) or urinary diversion with cystostomy, followed by excision primary anastomosis (EPA) urethroplasty. While PER has a low success rate (9 %), EPA urethroplasty demonstrates success rates of 90–95 % and is considered the standard of care for most PFUI-related strictures.^5^ If PER fails, definitive treatment should proceed with urethroplasty.^6^^,^^7^

PFUI can cause urinary tract obstruction, extravasation, and secondary sepsis acutely, and can also lead to strictures, erectile dysfunction, and incontinence, all of which can be associated with lifelong disability.^8^ Re-do urethroplasty poses challenges due to fibrosis, deep stricture location, and patient anxiety over outcomes, especially regarding sexual function.^9^^,^^10^

We present a case of complex urethral stricture following multiple failed interventions, complicated by a prostatorectal fistula. Surgical management was achieved with re-do EPA urethroplasty and gracilis muscle flap for fistula repair and vascular support.

## 2 Case presentation

A 32-year-old male presented with inability to void for the past nine years following a motorcycle accident in 2012. Between 2012 and 2021, he underwent six procedures: orthopedic and digestive surgeries (twice), endoscopic realignment, internal urethrotomy, urethral dilatation, and EPA urethroplasty. An iatrogenic prostatorectal fistula developed after internal urethrotomy. Preoperative BVCUG showed a bulbar urethral stricture with visible prostatorectal fistula (Fig. 1A, Fig. 1BA-B).

**Fig. 1A:**
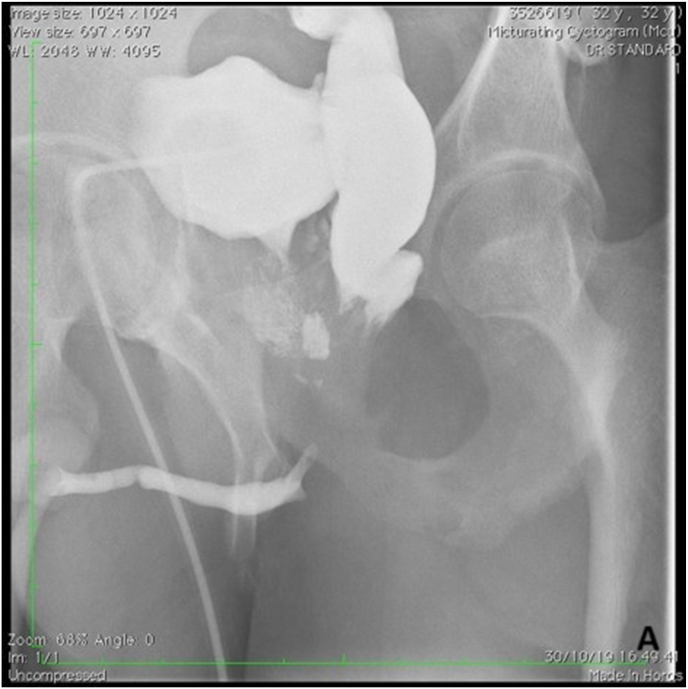
Preoperative BVCUG

**Fig. 1B:**
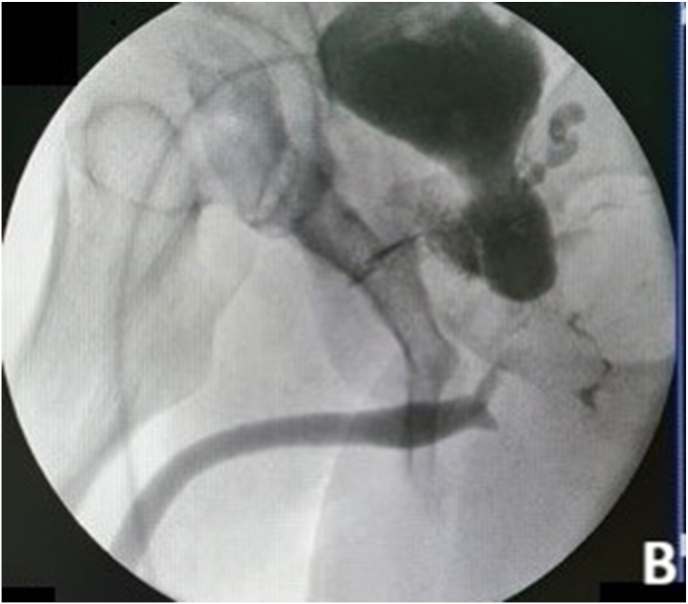
Preoperative BVCUG showing bulbar urethral stricture with prostatorectal fistula.

Re-do EPA urethroplasty and gracilis muscle flap interposition were planned. Under lithotomy position, an inverted U perineal incision was made (Fig. 2A). Panendoscopy confirmed the presence of a prostatorectal fistula (Fig. 2B). A Lone Star® retractor was placed to widen the operative field. The bulbar urethra was carefully mobilized up to the penoscrotal junction (Fig. 2C), and fibrotic tissue was excised following transection (Fig. 2D). To access the posterior urethra, the penile crura were separated (Fig. 2E), and an inferior pubectomy was performed to reduce urethral tension (Fig. 2F).

**Fig. 2A:**
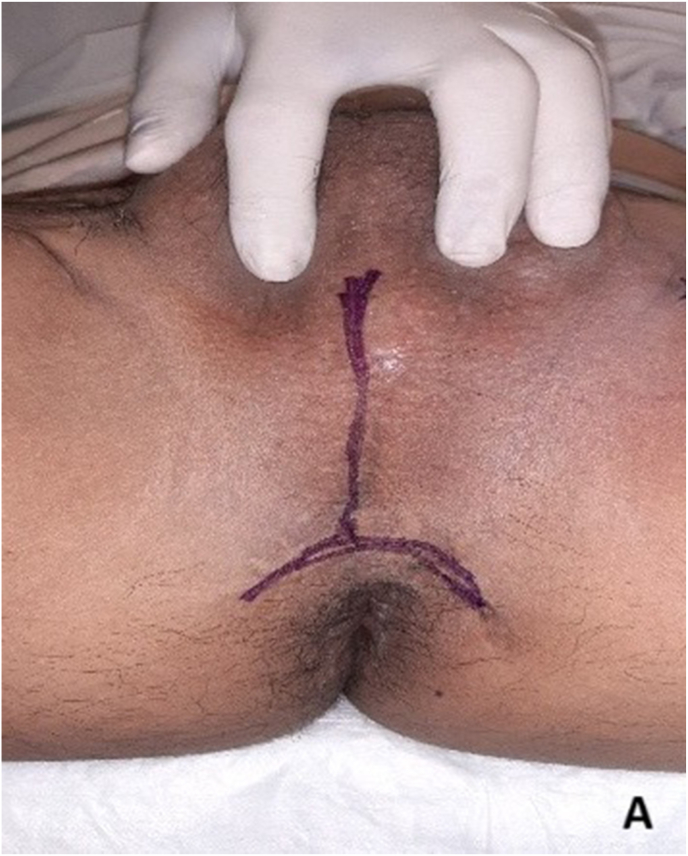
Intraoperative steps: Incision design.

**Fig. 2B:**
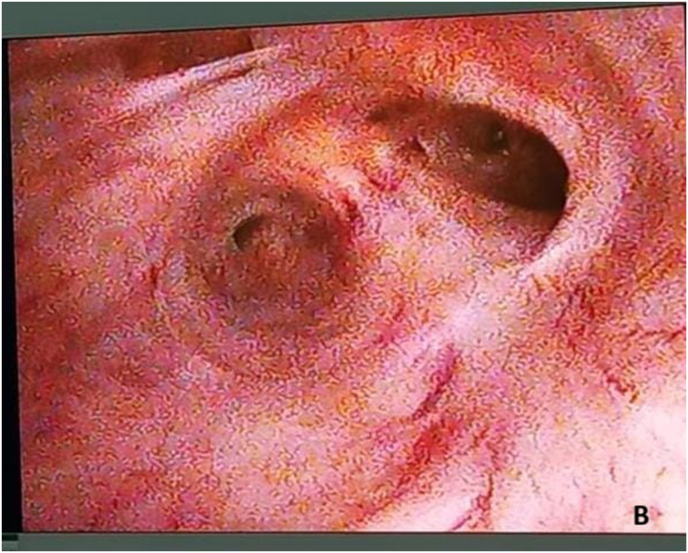
Intraoperative steps: Panendoscopy shows prostatorectal fistula.

**Fig. 2C:**
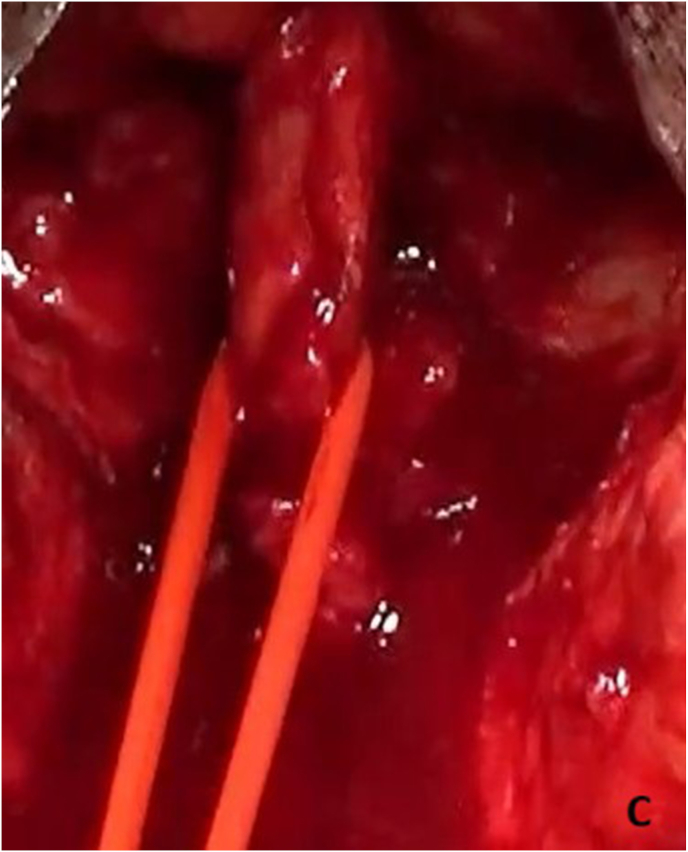
Intraoperative steps: Bulbar mobilization.

**Fig. 2D:**
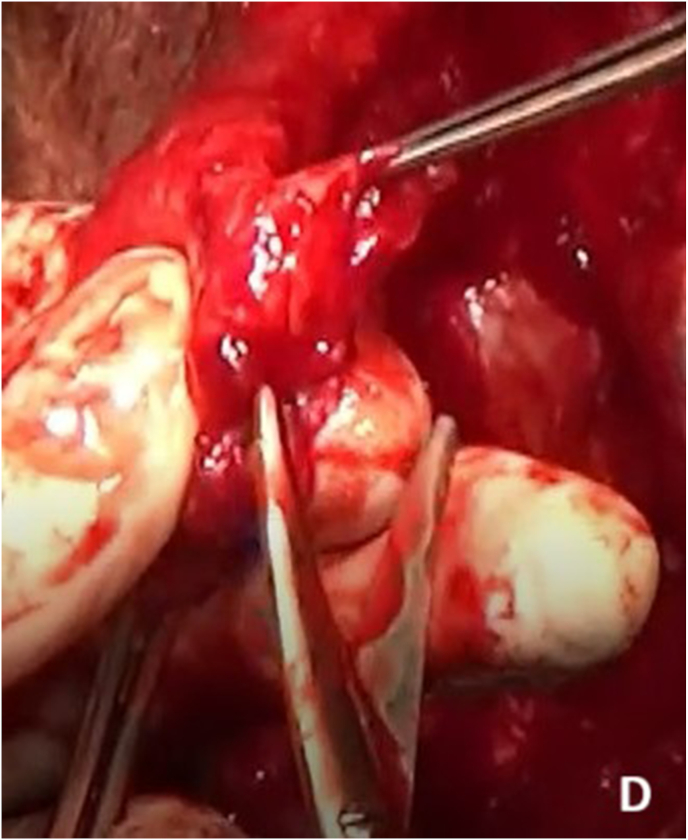
Intraoperative steps: Fibrotic tissue excision.

**Fig. 2E:**
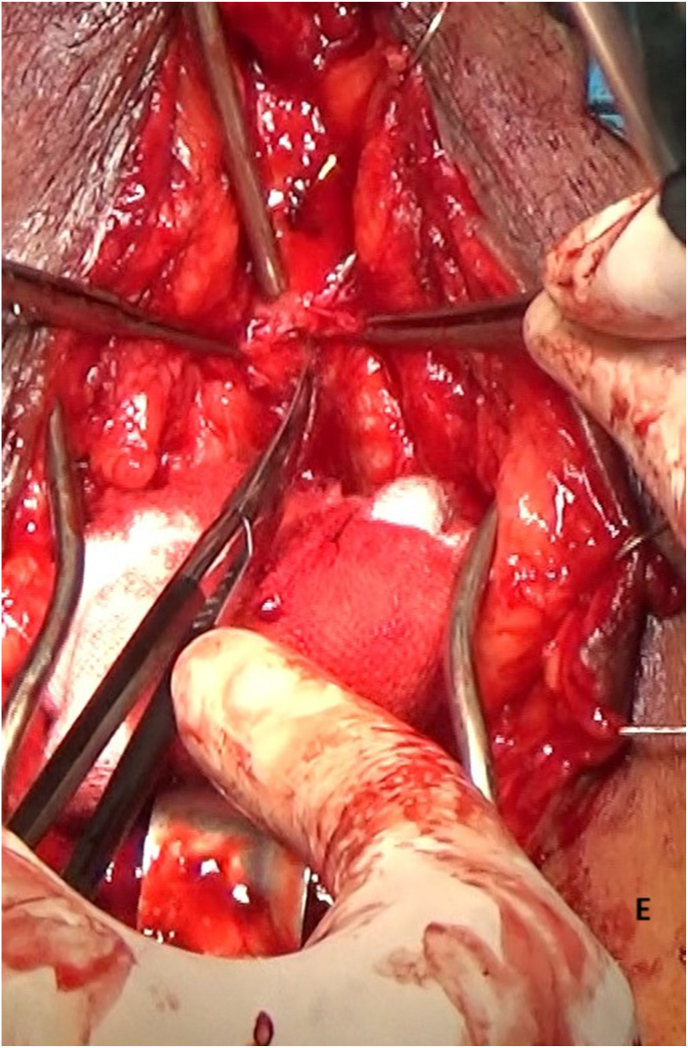
Intraoperative steps: Penile Crural Separation.

**Fig. 2F:**
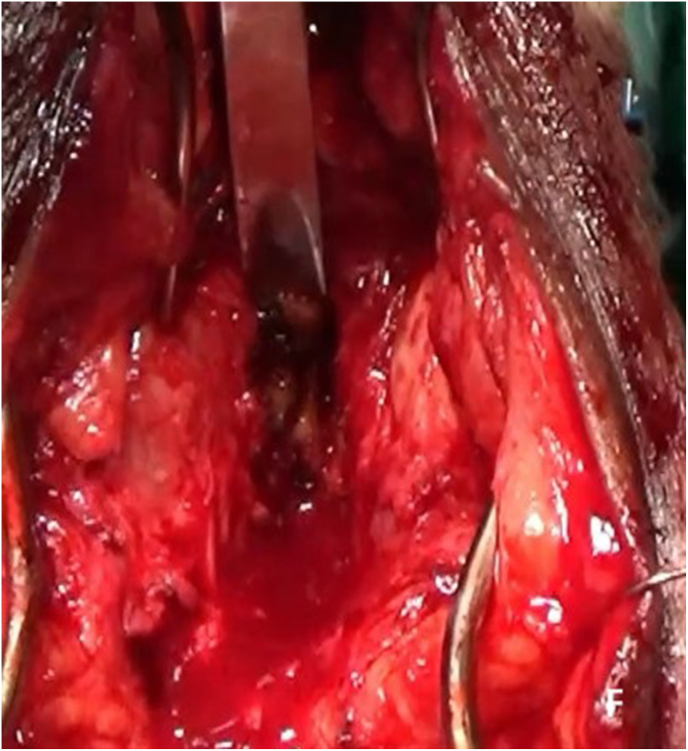
Intraoperative steps: Inferior pubectomy.

The prostatorectal fistula was identified by digital rectal examination and repaired in layers to minimize recurrence risk (Fig. 3A). The defect was closed, followed by panendoscopy to confirm the repair. A tension-free bulbo-membranous urethral anastomosis was completed (Fig. 3B). Gracilis muscle flap harvest was initiated by identifying its anatomical origin at the inferior ischiopubic ramus and insertion at the medial tibial condyle (Fig. 3C). The main pedicle of the gracilis muscle was located approximately 8–12 cm from its origin and preserved (Fig. 3D). A skin incision was made 2–3 fingers below this point. The muscle was then carefully mobilized distally while maintaining vascular integrity (Fig. 3E).

**Fig. 3A:**
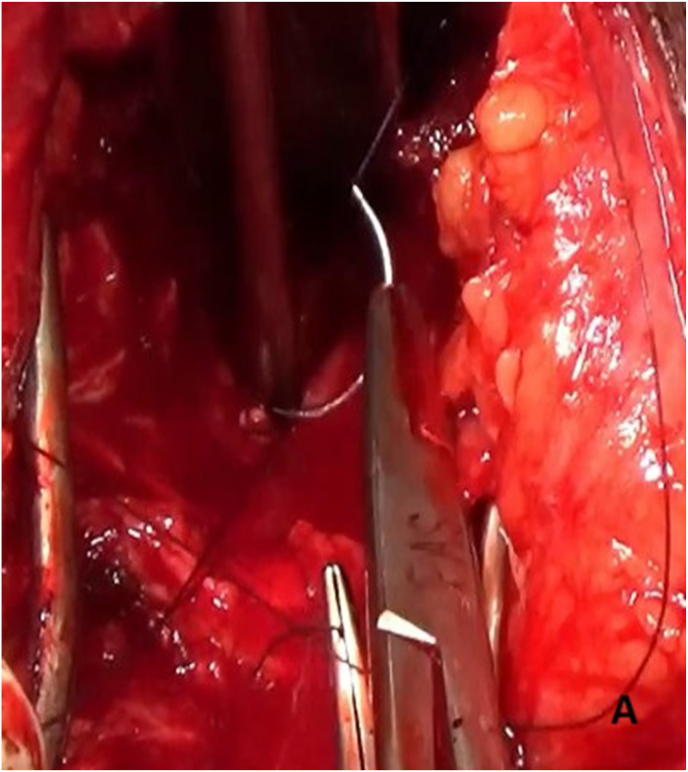
Intraoperative steps: Prostato rectal fistula repair.

**Fig. 3B:**
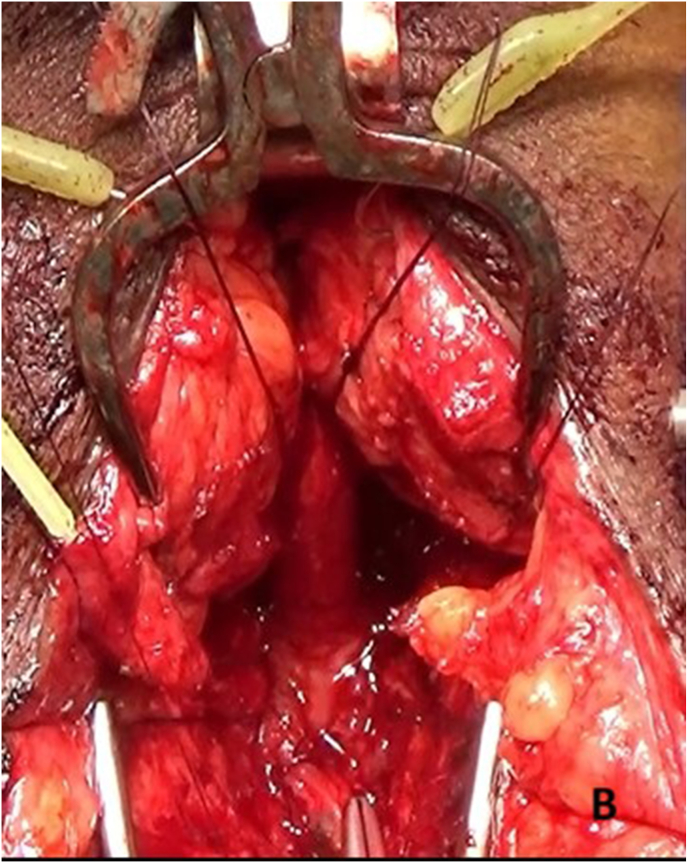
Intraoperative steps: Free tension anastomosis of Bulbomembranous Urethra.

**Fig. 3C:**
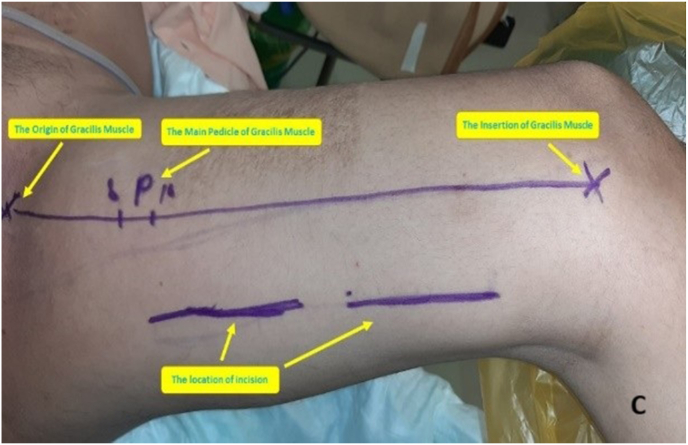
Intraoperative steps: Identification of Gracilis Muscle Flap.

**Fig. 3D:**
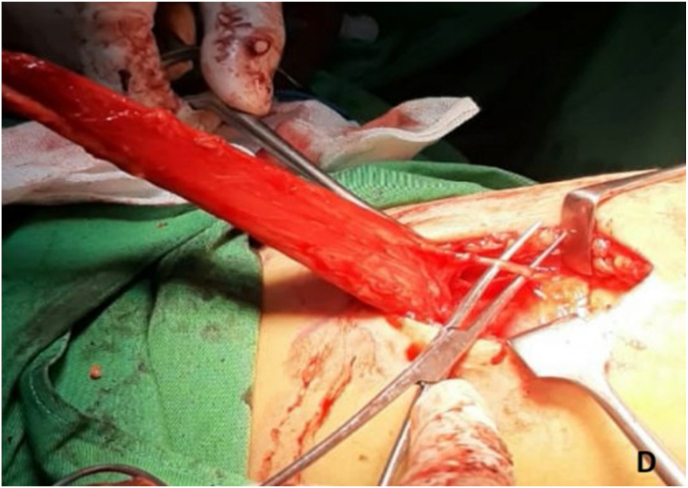
Intraoperative steps: Main pedicle of gracilis muscle flap.

**Fig. 3E:**
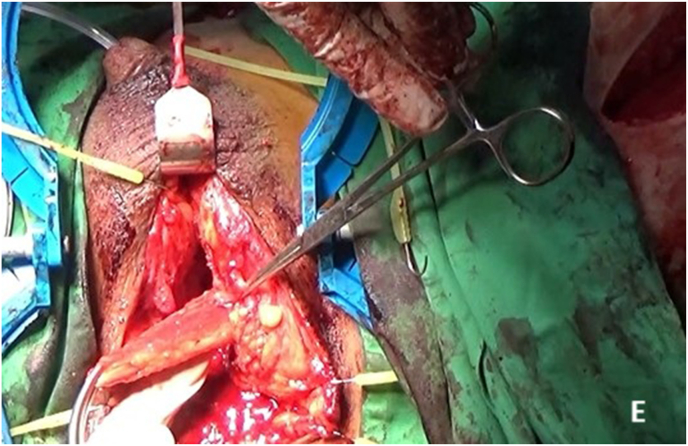
Intraoperative steps: Mobilization of gracilis muscle flap.

A subcutaneous tunnel was created to pass the gracilis flap into the perineum. The muscle was interposed between the urethra and rectum to reinforce the fistula repair and provide additional neovascular support (Fig. 4A). The immediate postoperative condition was favorable, with a clean perineal field and no signs of complications (Fig. 4B).

**Fig. 4A:**
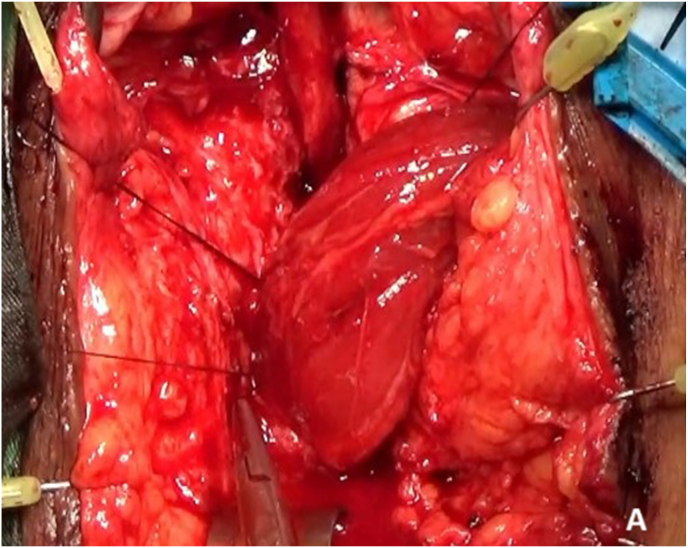
Intraoperative steps: Gracilis muscle flap.

**Fig. 4B:**
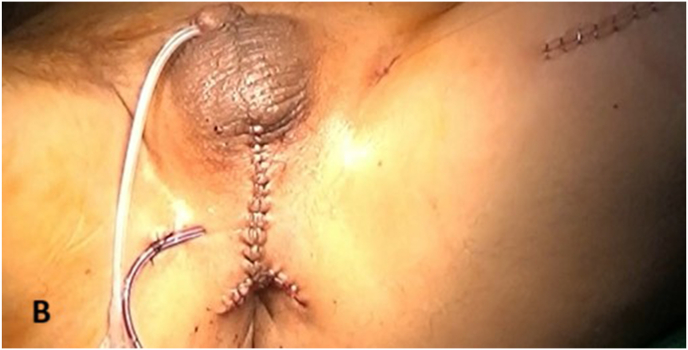
Post operative condition.

A 16-Fr Foley catheter was maintained for six weeks. Postoperative evaluation showed a Qmax of 14.8 mL/s on uroflowmetry (Fig. 5A) and good urethral patency on urethrography (Fig. 5B). The patient reported spontaneous voiding and satisfactory urinary function during six months of follow-up.

**Fig. 5A:**
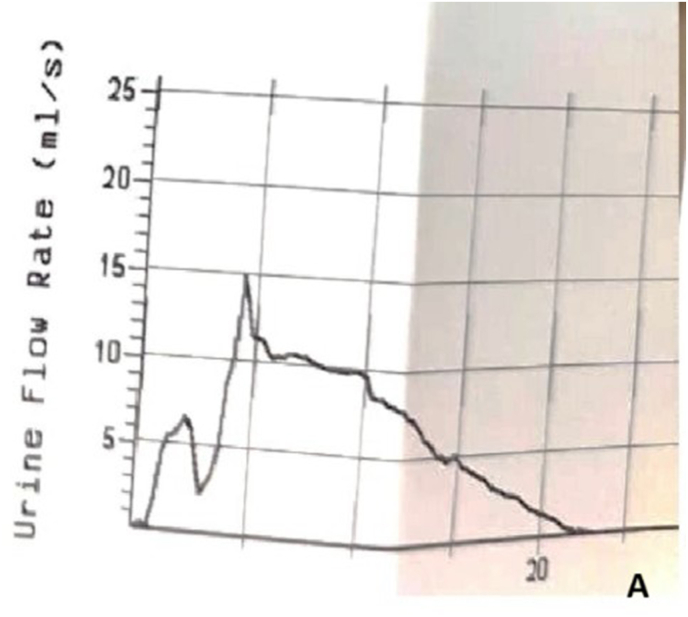
Post operative uroflowmetry and urethrography.

**Fig. 5B:**
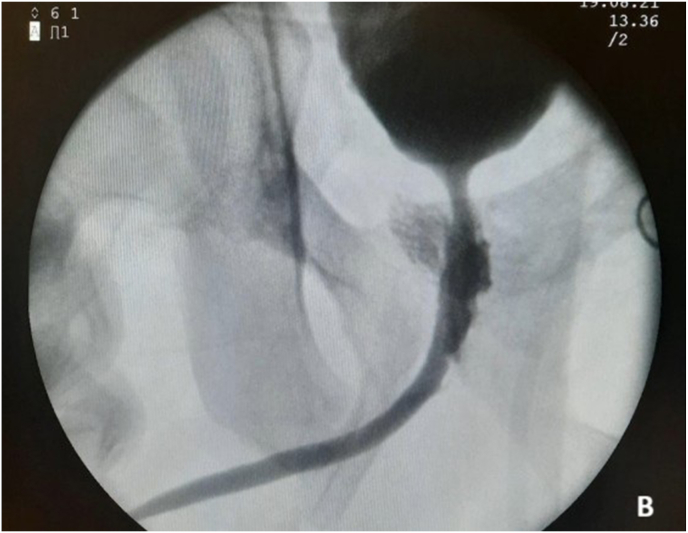
Post operative uroflowmetry and urethrography.

## 3 Discussion

The principal aim of urethroplasty in PFUI is complete excision of scar tissue and tension-free anastomosis of healthy urethra. Substitution urethroplasty using flaps or grafts is rarely needed unless there is a long gap or inadequate urethral length.^11^ Recurrent strictures often result from ischemia due to vascular injury, tension at the suture line, or inadequate excision of fibrosis.^12^

In this case, the patient experienced multiple failed interventions and developed a rare complication, prostatorectal fistula. Gracilis muscle flap was selected to support healing and provide vascularization. Palmer et al. report an 80 % success rate using gracilis flap in high-risk reconstructions.^13^ The flap offers neovascular support, a physical barrier for fistulas, and an easily accessible donor site. Gracilis flaps have demonstrated benefits in infected, ischemic, or previously operated fields.^14^

The gracilis muscle is especially advantageous due to its reliable pedicle, ease of harvest, and arc of rotation into the perineum without significant morbidity. It has been used to reinforce urethral repairs, cover buccal mucosal grafts, or even create neourethras when tubed with skin components.^15^^,^^16^

The urethra receives dual vascular supply: antegrade flow from the bulbar and circumflex cavernosal arteries and retrograde flow from the dorsal penile artery. This redundancy enables safe transection and anastomosis.^15^ Nonetheless, pelvic trauma may disrupt pudendal vessels, compromising urethral blood flow and healing. Revascularization in such cases improves surgical success rates.^17^

Various surgical strategies have been described depending on stricture location, length, etiology, and prior interventions. For short bulbar strictures, the International Consultation on Urologic Diseases (ICUD) recommends EPA with a composite success rate of 93.8 %.^17^ For strictures less than 2 cm in length, excision and primary anastomosis (EPA) has shown excellent long term results.^18^ Although EPA is recommended for strictures shorter than 2 cm, Demir et al. showed that it can be used for strictures of up to 5 cm. In patients with long urethral strictures the corpus cavernosum can be split (corpora separation) to relieve urethral tension by shortening the distance between the two ends. However, if urethral tension persists after splitting, the distance can be shortened by removing the inferior portion of pubis.^19^ Kulkarni et al. shows success rate of urethroplasty with inferior pubectomy as a management of PFUI is 89 %.^20^

However, despite its high success rates, EPA is considered to increase the risk of sexual dysfunction.^19^ But, in recent study, Hults reported a significant decline in the IIEF-5 score at the first follow-up in sexually active patients who had good erectile function before surgery. This first decline could be attributed to pain and catheterization during the first weeks after surgery. Full recovery of erectile function was seen at the third follow up. In the similar study, EPA led to a significant decrease in IPSS score, as measured at the first, second, and third follow-up visits, indicating an improvement in LUTS.^16^

## 4 Conclusion

Re-do EPA urethroplasty combined with gracilis muscle flap provides a viable solution for managing complex urethral strictures with associated complications such as prostatorectal fistula. The use of gracilis flap offers vascular support, promotes healing, and reduces the risk of recurrence. Our case demonstrates favorable functional outcomes and supports the application of this technique in select high-complexity cases.

## CRediT authorship contribution statement

**Kristian Mohamad Daniputra:** Conceptualization, Data curation, Formal analysis, Funding acquisition, Investigation, Methodology, Project administration, Resources, Software, Validation, Visualization, Writing – original draft. **Paksi Satyagraha:** Supervision, Validation, Writing – review & editing. **Pradana Nurhadi:** Supervision, Validation, Writing – review & editing.

## Funding

This research did not receive any specific grant from funding agencies in the public, commercial, or not-for-profit sectors.

## Declaration of competing interest

The authors declare that they have no known competing financial interests or personal relationships that could have appeared to influence the work reported in this paper.
